# Occurrence of Lymphatic Filariasis infection after 15 years of mass drug administration in two hotspot districts in the Upper East Region of Ghana

**DOI:** 10.1371/journal.pntd.0010129

**Published:** 2022-08-04

**Authors:** Derrick Adu Mensah, Linda Batsa Debrah, Peter Akosah Gyamfi, Abu Abudu Rahamani, Vera Serwaa Opoku, John Boateng, Prince Obeng, Jubin Osei-Mensah, Inge Kroidl, Ute Klarmann-Schulz, Achim Hoerauf, Alexander Yaw Debrah

**Affiliations:** 1 Department of Clinical Microbiology, School of Medicine and Dentistry, Kwame Nkrumah University of Science and Technology, Kumasi, Ghana; 2 Kumasi Centre for Collaborative Research in Tropical Medicine, Kwame Nkrumah University of Science and Technology, Kumasi, Ghana; 3 Faculty of Health Sciences, Garden City University College, Kumasi, Ghana; 4 Division of Infectious Diseases and Tropical Medicine, Medical Center of the University of Munich (LMU), Munich, Germany; 5 German Center for Infection Research (DZIF), Munich, Germany; 6 Institute for Medical Microbiology, Immunology and Parasitology, University Hospital Bonn, Bonn, Germany; 7 German Center for Infection Research (DZIF), Bonn-Cologne, Germany; 8 Faculty of Allied Health Sciences, Kwame Nkrumah University of Science and Technology, Kumasi, Ghana; Washington University School of Medicine, UNITED STATES

## Abstract

**Background:**

Lymphatic filariasis (LF) causes chronic morbidity, which usually manifests as lymphedema or hydrocele. Mass drug administration (MDA) began in Kassena Nankana East Municipal (KNEM) and Nabdam, two hotspot districts in the Upper East Region in Ghana, in 2000 and 2005, respectively. This cross-sectional study evaluated the impact of 15 years of MDA on the control of LF as determined by circulating filarial antigen (CFA) and microfilariae assessment in the KNEM and the Nabdam districts.

**Methodology/Principal findings:**

A total of 7,453 participants from eight sub-districts in the two hotspot districts (KNEM: N = 4604; Nabdam: N = 2849) were recruited into the study. The overall CFA prevalence as determined by the FTS was 19.6% and 12.8% in the KNEM and Nabdam districts, respectively. Manyoro, a sub-district on the border with Burkina Faso, recorded the highest CFA prevalence of 26% in the KNEM. Assessment of microfilariae and Og4C3 antigen was done from 1009 (KNEM: N = 799 (79.2%); Nabdam: N = 210 (20.8%)) randomly selected FTS-positive (N = 885) and FTS-negative (N = 124) individuals. The Og4C3 antigen was found in 22.6%/23.0% of the selected individuals (KNEM/Nabdam), whereas the night blood revealed microfilariae in only 0.7%/0.5%.

**Conclusions/Significance:**

Using the WHO endorsed FTS, CFA prevalence exceeded the long-standing <2% threshold—which may need revision and validation. Surprisingly, the Og4C3 ELISA showed positive results in only about one-fifth of the FTS positive samples. However, even this result would not have met the <2% CFA criteria for LF elimination. In contrast, projections from the microfilariae results revealed a halt in LF transmission. The global elimination target was due in 2020 but has been extended to 2030 since this could not be met. Focused MDA intervention intensification on seasonal migrants and non-compliers, and implementation of alternative treatment strategies may suffice for the elimination of the disease.

## Author summary

Lymphatic filariasis (LF) is a major neglected tropical disease (NTD) affecting over 120 million individuals worldwide and identified as one of WHO’s 20 NTDs targeted for elimination. It has strong links with poverty and is associated with significant clinical morbidity, which impose considerable socio-psychological and economic burdens on the affected individuals. One of the main goals of the Global Programme to Eliminate Lymphatic Filariasis (GPELF) is the use of mass drug administration (MDA) to interrupt LF transmission. The initial 2020 elimination target year set by the GPELF could not be met, with 2030 now the new proposed year targeted for global elimination. The study evaluated the impact of 15 years of MDA on the control of LF in two hotspot districts in Ghana. The results from this study are indicative of a halt in LF transmission in the districts, with microfilaria detected in <1% of those sampled. However, the antigenemia prevalence is still above the recommended level. MDA should be intensified especially at the border towns or alternative treatment strategies should be employed to finally eliminate the disease as some people who are still with the active infection can serve as a reservoir for recrudescence and transmission in already “LF-free” areas.

## Introduction

Lymphatic filariasis (LF) is a disease of public health importance in low and middle-income countries in Asia, Africa, Western Pacific, the Caribbean and South America. Recent global estimates indicate that the infection is endemic in 50 countries and there are about 859 million people at risk of the infection and requiring Mass Drug Administration (MDA) and of these, over 343 million (39.9%) are found in Africa [1]. At least 36 million people remain burdened with the debilitating chronic disease manifestations associated with LF (i.e., hydrocele and lymphedema).

LF is caused by three species of parasitic filarial nematodes *Wuchereria bancrofti*, *Brugia malayi and Brugia timori* with *W*. *bancrofti* accounting for over 90% of all infections [2]. The disease is transmitted through the bite of an *Anopheles* or *Culex* mosquito carrying the third stage larval infective form of *W*. *bancrofti* and *Brugia spp*. respectively. Adult worms of these parasites have an affinity for the lymph vessels and lymph nodes in humans and can live for about four to six years in these sites. Infections most commonly result in clinical manifestations of the disease such as hydrocele, lymphedema, chyluria and episodic attacks of painful adenolymphangitis attacks (ADLA) [3]. The disease greatly impairs the physical, social and mental well-being due to the severe social stigma associated with it [1,2]. In an effort to tackle the disease, the Global Alliance to Eliminate Lymphatic Filariasis (GAELF) was created (as a platform supported by filarial endemic country governments and NGOs) in 2000 with the aim of eliminating LF by the year 2020. The Global Programme to Eliminate Lymphatic Filariasis (GPELF) was set up with the main goal of eliminating LF through the use of MDA programs in endemic areas and also to provide and implement measures that aid in managing morbidity associated with chronic cases of LF [2]. The program provides free albendazole with either ivermectin or diethylcarbamazine (DEC) for distribution in filarial endemic countries. The use of DEC is however discouraged in areas that are co-endemic for Onchocerciasis due to potential side effects including blindness [4]. The GPELF has been a hugely successful programme thus far with impressive progress having been made; so far, 7.7 billion MDA treatments have been provided with over 890 million people having taken MDA drugs at least once [1].

One of the key elements of the MDA programme to eliminate LF in a district is the regular assessment of its impact in reducing transmission of LF [5,6]. This is particularly important in determining end points for stopping an MDA programme in a community. When GPELF was started, five to six rounds of MDA covering at least 65% of a population in an endemic area was thought to be sufficient to reduce the CFA prevalence to less than 2% or MF prevalence to less than 1% and thereby halt transmission. The pre-Transmission Assessment Survey (pre-TAS) was the standardized method of LF prevalence testing adopted by the WHO [1,3]. However, this extrapolation has been questioned more recently [6] and may need a revision/adjustment or a validation especially after more sensitive diagnostic kits have been introduced and made available.

MDA was started in Ghana in 2000 and by 2006 all 98 filarial endemic districts were treated at least once, including the two hotspot districts currently being assessed in this present study [7]. Five years after the programme had started, prevalence of LF in some of these districts had been reduced by as much as 70% [8]. However, LF infection persists in some communities in Ghana despite having undergone more than 15 years of directly observed (the ideal but may not have been always followed by community drug distributors) MDA treatment reportedly covering over 65% of the endemic population. These districts, which include the Kassena Nankana East Municipal (KNEM) and Nabdam, are therefore termed as hotspots. As of 2017, there were still 22 of such ‘hotspot’ districts in Ghana [7]. The KNEM was one of the first municipalities to have started MDA in Ghana in 2000 while the Nabdam district started in 2005. According to Biritwum *et al*., (2017) the prevalence of CFA using the immunochromatographic test (ICT) card and MF positive individuals in the KNEM district had reduced from 45.4% and 29.6% at baseline in 2000 to 6.6% and 4.0%, respectively, in 2007 [7]. Data on the current prevalence of LF in the KNEM and Nabdam districts is scanty, obsolete and not robust; as very few LF prevalence assessments have been done in the districts. Also, whilst 2020 was the initial target year set by GPELF for elimination, 2030 has now been proposed as the new target year for elimination since the initial target was not met [9]. Delays were attributed to the issue of non-compliance, logistics in endemic countries and the fact that ivermectin (IVM) cannot be distributed in areas (Central and West Africa predominantly) co-endemic for another filarial nematode, *Loa loa*, due to the risk of severe adverse events, especially toxic encephalopathy [9].

There has been the need for a study to assess the current prevalence of LF (both MF infected and those suffering from pathology) in the KNEM and Nabdam districts, which are two of the hotspots in Ghana to ascertain the progress made in terms of CFA and MF reduction towards LF elimination. However, due to the primarily microfilaricidal activity of the current drug regimen used for MDA—with limited effect on the adult worms, most sentinel sites show distinct antigen and MF prevalence as typically low MF prevalence but relatively high antigen prevalence after several years of MDA [6]. Considering the high sensitivity, and also the advantage of using the Tropbio Og4C3 antigen ELISA kits and the Alere Filariasis Test Strip (FTS) with day blood samples, the utility of these immunological markers in participant antigen detection, treatment monitoring and post-treatment surveillance is recommended [3].

A recent study has indicated an increased risk of pre-TAS failure when the FTS is used to assess treatment impact on antigenemia levels among adults [5]. Moreover, though the FTS is endorsed by WHO, there is an unresolved concern about the accuracy of the FTS in detecting active bancroftian infection due to the persistence of residual antigens after adult worm death and also particularly due to the test’s inadequate specificity for antigens from other filarial nematodes [10,11] such as *L*. *loa*, *Onchocerca volvulus* and *Mansonella perstans*—with the last two being co-endemic with LF in Ghana [12–14]. Thus, it remains uncertain as to whether the estimated CFA prevalence in endemic areas with higher MDA rounds is reflective of the true burden of active LF infection. To address these concerns, we envisaged the evaluation of the test performance of the FTS and the alternative Og4C3 TropBio Enzyme-Linked immunosorbent assay (ELISA) CFA test—which in addition provides, the quantitative measures of CFA used as a proxy to monitor the progress of antifilarial treatment. We however hypothesize that, as in most sentinel sites the prevalence is expected to be typically low for MF but relatively high for antigen after several years of MDA. The aim of this study was to assess the impact of 15 years of Mass Drug Administration on Lymphatic Filariasis control efforts in the Kassena Nankana East Municipal and the Nabdam districts as determined by CFA assessment using the Alere Filariasis Test Strip (FTS) and the Og4C3 Tropbio ELISA as well as MF assessment using night blood samples.

## Methods

### Ethics statement

Ethical approval was obtained from the Committee on Human Research Publication and Ethics (CHRPE) of the School of Medicine and Dentistry and Kwame Nkrumah University of Science and Technology (KNUST). Permission was also obtained from village opinion leaders and community elders prior to commencement of the study. Approvals were also obtained from Kassena Nankana East Municipal, the Nabdam, and Upper East Regional Health Directorates. Further ethical approval was obtained from Bonn University Hospital Ethical Committee. Participants were educated and informed about the various processes that would be carried out in the screening and the purpose of the study. Written consent was obtained from all adult participants either by thumbprinting or signing. Assents were completed for participants who were under 18 years, with parents/guardians giving formal written consent for such volunteers. This study was part of other larger studies namely: “Tackling the Obstacle to Fight Filariasis and Podoconiosis” (TAKeOFF)- sponsored by German Ministry of Education and Research (BMBF), the First Stage Genome Wide Association study of Lymphatic Filariasis pathology (GWAS)- sponsored by the German Research Foundation (DFG) and the Alternative treatment Strategies using anti-wolbachial drugs to accelerate elimination of Lymphatic Filariasis and Onchocerciasis (ASTAWOL)- sponsored by EDCTP. The ethical approval number/codes for these studies are CHRPE/AP/525/17; CHRPE/AP/128 and CHRPE/AP/337/20.

### Study area/setting, site and population

The study was carried out in the Kassena-Nankana East Municipal and the Nabdam districts of the Upper East Region of Ghana. These areas have an annual rainfall of approximately 100 cm, most of which falls between May and September with temperatures ranging between 16°C and 41°C. The area is contiguous with the border of Burkina Faso, Bolgatanga Central, Builsa North and South districts and the Bawku West Municipalities and has a typical Sahelian ecological setting. It is mainly rural with dispersed settlements of extended family compounds surrounded by their farm lands. Less than 20% of the community has had any formal education and they are materially very poor [15]. There are numerous small dams in the area, and consequently, many favourable breeding sites for mosquitoes exist. There are three main ethnic groups in the two districts, the Kassenas and Nankanis with a minority Builsas in the KNEM: with the Nabt ethnicity dominating the Nabdam district [15]. The study was conducted in 28 LF endemic communities in three sub-districts of Kassena-Nankana East Municipal (Pungu, Navrongo-East and the Manyoro sub-districts) and 33 LF endemic communities in five sub-districts (Nagodi, Pelungu, Sakote, Zanlerigu and Kongo-Pitanga) in the Nabdam district. The KNEM was one of the first municipalities to have started MDA in Ghana in 2000 while the Nabdam district started in 2005 [7,8]. According to Biritwum *et al*., (2017) the prevalence of CFA using the ICT and MF positive individuals in the KNEM district had reduced from 45.4% and 29.6% at baseline in 2000 to 6.6% and 4.0%, respectively, in 2007 [7,8]. There has however not been any LF prevalence work done since MDA started in the Nabdam district.

### Study design

A cross-sectional study from June 2018 to May 2021 with a purposive community sampling approach was employed. Some communities were not motorable/reachable by vehicle so only motorable communities were selected for screening. The study was part of three larger studies as stated above. A few days prior to the recruitment, announcements were made via the community information centers or by a ‘gong-gong’ beater (a local means of giving information to community residents where the ‘beater’ moves around sounding a metallic instrument ‘the gong gong’ whilst intermittently shouting out the information). Any individual ≥5 years who had stayed in the endemic community for ≥2 years and is mentally sound and can give consent (for adults) or consent on behalf of their wards (minors); was deemed fit for inclusion. The exclusion criteria included any individual <5 years who had stayed in the endemic community for <2 years, not mentally sound and not ready to give consent. Community residents were informed by the community health volunteers to gather at chosen social centers. The study was explained in English and then in the local languages ‘Kassim, Nankani and Nabt’ during the meeting with the community elders and interested people at a chosen gathering point in the community. The participants were invited to ask questions and these were answered. During the surveys, persons identified as eligible to participate in the study were informed individually in English by the research team and then in ‘Twi’, “Nabt, “Kassim” or “Nankani” as preferred by the participant. Informed, signed/thumb printed, or witnessed consent was obtained from all participants. The Informed Consent Forms were either in English, “Twi”, “Nabt”, “Kassim” or “Nankani”.

### Recruitment of study participants and data collection

A total of 4604 participants from KNEM and 2849 from the Nabdam district, aged 5 years and above were recruited for the study. According to the district disease control officers of the two hotspot districts, all communities screened had completed ≥15 MDA rounds and the last rounds were carried out in 2016 and 2019 in the KNEM and the Nabdam district, respectively. Participants were identified using a Participant Identification Number (PID) consisting of unique village codes and screening numbers. Demographic data of all consenting participants comprising age, sex, number of IVM rounds taken, household name, occupation and number of years living in LF endemic community were recorded. This was assessed via participant interviewing, with interviews structured according to a predefined questionnaire which was administered by the investigators (research team) at a place far from the gathering, with participants assured of data safety and confidentiality of their responses. To prevent MDA intake/treatment rounds recall bias, responses from participants were re-checked/cross-checked from the treatment records with the community health volunteers in the communities who have the MDA records for every household. This was done before the numbers were used in any analyses. Participants in the study communities were then screened for CFA using the Alere Filarial Test Strip (FTS) (Alere, Scarborough, ME) read in 10 minutes independently by two investigators following the manufacturer’s instructions. From all the participants, fresh capillary blood samples obtained from finger pricking in the communities were used for the FTS test. Because the MF of the *W*. *bancrofti* parasite is nocturnally periodic [1–3], night blood samples (between 10 pm-12:00 am) were collected from randomly selected FTS+/- participants for further analysis.

### Examination of participants with LF pathologies (lymphedema and hydrocele)

Participants who came for the screening presenting with non-traumatic progressive and evolving swelling of a limb, intermittently experiencing the acute stages/attacks of the disease (ADLA) and depicting the overt manifestations of the disease (lymphedema and hydrocele) were physically screened based on medical history and physical assessment. Study participants with LE stages 1–2 were considered to have mild LE, stages 3–4 had moderate LE and stages 5–7 had severe LE [16]. Male participants were further asked if they had scrotal swelling. A study clinician was responsible for the identification and confirmation of the hydrocele cases.

### Sample collection

Night blood samples (⁓5 mL) were collected from randomly selected FTS+/- participants by trained phlebotomists between the hours of 10 pm-12:00 am from the median cubital superficial vein of the upper limb. Sterile disposable hypodermic vacutainer needles were used for the venipuncture and blood was collected into EDTA tubes for the assessment of MF (using whole blood sample) by microscopy and Og4C3 antigen (plasma sample) using the Tropbio ELISA. After 2 mL of whole blood has been aliquoted, ⁓1 mL of plasma were collected from the remaining whole blood samples of each participant into 1.5 mL Eppendorf tubes after centrifuging at 2000g for 10 minutes and preserved at -20°C for later Og4C3 antigen analysis at the Kumasi Centre for Collaborative Research in Tropical Medicine (KCCR), KNUST-Kumasi.

### Laboratory investigations

#### Sedgewick and Giemsa/Filter counts

Randomly selected participants who were FTS positive (694/191) and negative (105/19) in KNEM/Nabdam respectively were screened for MF and its quantification, using 2mL of the night venous whole blood samples collected into the EDTA tubes. Two hundred microliters (200 μL) of the blood sample were diluted in 800μl of 3% acetic acid and transferred into a Sedgewick counting chamber. A confirmatory test was done by filtering 1 mL of whole blood mixed with 8 mL of distilled water through a 5 mm Whatmann Nucleopore filter and then fixed with methanol on a microscope slide. The Filter was air dried, stained with 5% Giemsa and observed under the X10 objective lens of a light microscope. The number of MF counted were recorded as MF/mL.

#### Og4C3 Tropbio Enzyme Linked Immunosorbent Assay (Og4C3 Tropbio ELISA)

The Og4C3 ELISA test kit, specific for *W*. *Bancrofti* CFA, was used following the manufacturer’s recommendations (TropBio; Cellabs, Dale Street Brookvale, NSW 2100 Australia; LF2.3; KF1/KF2). Hundred microlitres (100 μL) of each individual’s plasma sample was used for the Tropbio ELISA test (Og4C3 antigen assessment) as directed by the manufacturer. Microtitre plates were examined using a spectrophotometer (SpectraMAX 190) at 450/620 nm.

#### Og4C3 ELISA assay results interpretation

According to the manufacturer’s updated recommendation (August, 2021) for areas which have had many rounds of MDA and has resulted in low LF prevalence; standard 2 should be used as a reference for determining a cut-off value to define a low positive and a negative sample (TropBio; Cellabs, Dale Street Brookvale, NSW 2100 Australia; LF2.3; KF1/KF2). If standard 2 OD is within +/- 10% of 0.35, this value was used as the cut-off for result interpretation. However, for ELISA plates with Standard 2 OD not within range (+/- 10% 0.35), a default cut-off OD of 0.35 was used. Thus, the assay interpretation was set at; Negative = any OD below the set cut-off; Low Positive = any OD between 0.8 and the set cut-off. Medium Positive = OD between 0.8–1.0 and High Positive = OD >1.0.

#### Statistical analysis and plan

Data entry was done using Microsoft Office Excel 2010. Data were analysed for the prevalence of antigenemia (CFA) and MF across a number of demographic parameters and number of MDA rounds. Test of association among variables and infection status (CFA and MF) were done using Pearson-Chi-square test on GraphPad-Prism version 6.02. For non-parametric data sets, analysis was done using the Mann-Whitney U test. For determination of statistical trends in age and LF infection status, the Cochran-Armitage test for trend was used. Statistical significance was set at p<0.05, with confidence interval at 95%. Risk of infection among gender was analysed using odds ratio (OR) in Statistical Package for Social Sciences (SPSS) version 23 software.

All reported prevalence are prevalence estimates based on the voluntary participation in the study and not an epidemiological pre-defined sample.

## Results

### Demographic distribution and CFA prevalence of study participants as determined by FTS

**Fig 1:**
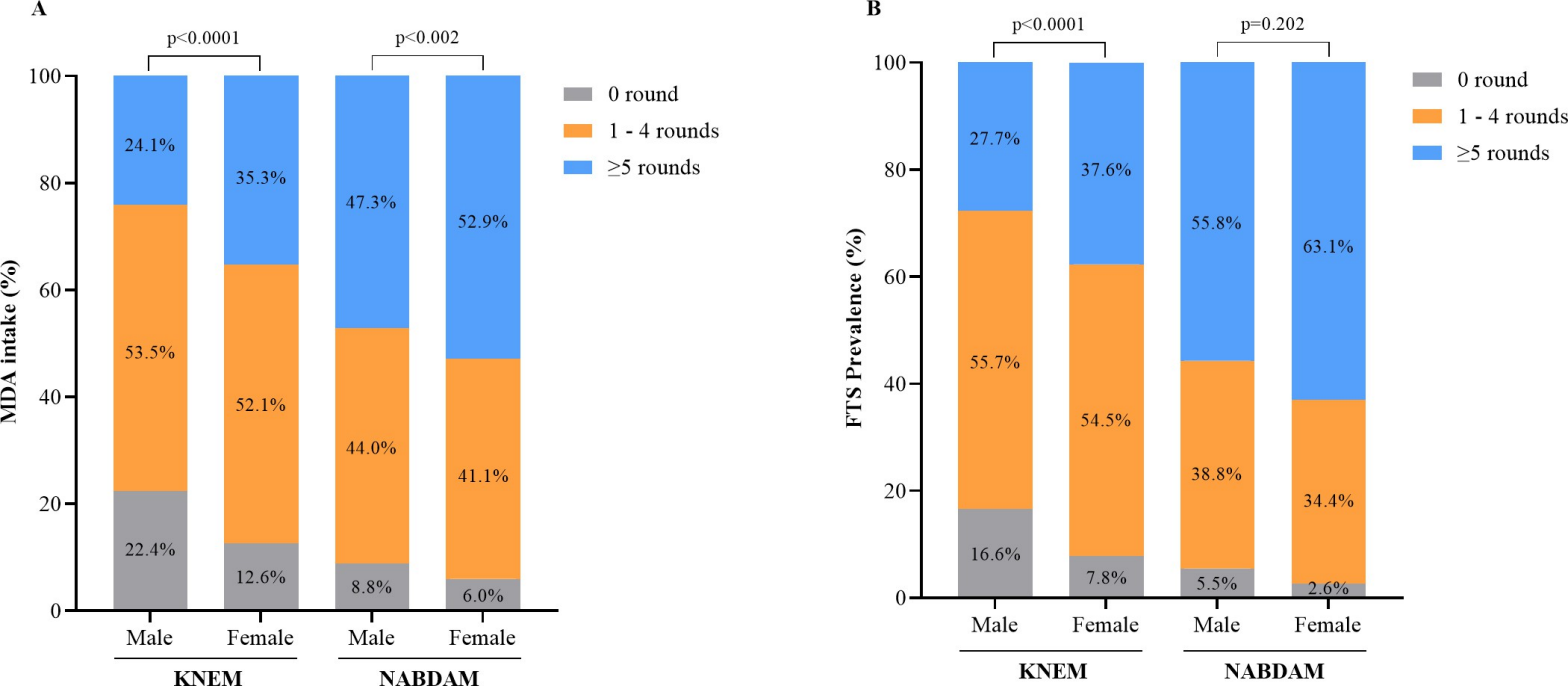
Gender distribution, MDA participation and FTS-CFA prevalence of study participants in the KNEM and Nabdam districts.

Out of the 4604 participants from KNEM who took part in the study, 2997 (65.1%) were females (Table 1). Close to 20% (904; 19.6%) of the participants were CFA positive as determined by the Alere FTS with a higher proportion of men being positive than females (21.3% vs. 18.7%; p = 0.033) (Table 1). Also, the age of the participants ranged from 5 to 91 years with a median of 38 years. In both districts, a smaller prevalence was found in the younger age groups and the rate increased significantly with age in both districts (p<0.001) (Tables 1 and 2). Manyoro, a sub-district on the border with Burkina Faso recorded the highest CFA prevalence of 26.01%. A total of 936 (12.6%) participants [738 (16%) in KNEM; 198 (7.0%) in Nabdam] had never taken part in the annual MDA rounds—with 10% and 17.2% being females and males respectively in both districts (Table 1 and Fig 1). There was a significant association (p<0.001) between FTS-CFA prevalence, gender and number of MDA rounds received in KNEM (p<0.0001) (Fig 1) but not in Nabdam (p<0.202).

**Table 1:** Demographic distribution and FTS prevalence of study participants in KNEM and Nabdam districts.

Variable	KNEM			Nabdam		
	Total screened	FTS positives	p-Value	Total screened	FTS positives	p-Value
**Age (years)** ^**a**^	38.0 (31)	44.0 (29)	**< 0.001** ^**b**^	38.0 (29)	43.0 (25)	**< 0.001** ^**b**^
**Years lived in endemic community** ^**a**^	34.0 (30)	41.0 (27)	**< 0.001** ^**b**^	35.00 (27)	40.0 (25)	**< 0.001** ^**b**^
**Gender** ^**c**^			**0.033** ^**d**^			**0.001** ^**d**^
Female	2997 (65.1%)	561 (18.7%)		1060 (37.5%)	165 (15.6%)	
Male	1607 (34.9%)	343 (21.3%)		1763 (62.5%)	195 (11.1%)	
**Total**	**4604 (100.0%)**	**904 (19.6%)**		**2823 (100.0**%**)**	**360 (12.8%)**	
**Age group (years)** ^**c**^			**< 0.001** ^**e**^			**< 0.001** ^**e**^
5 –≤10	318 (6.9%)	9 (2.8%)		2 (0.1%)	0 (0.0%)	
11–20	708 (15.4%)	55 (7.8%)		430 (15.2%)	29 (6.7%)	
21–30	792 (17.2%)	155 (19.6%)		577 (20.4%)	68 (11.8%)	
31–40	772 (16.8%)	167 (21.6%)		548 (19.4%)	70 (12.8%)	
41–50	745 (16.2%)	175 (23.5%)		465 (16.5%)	75 (16.1%)	
51–60	548 (11.9%)	149 (27.2%)		398 (14.1%)	69 (17.3%)	
>60 years	721 (15.7%)	194 (26.9%)		403 (14.3%)	49 (12.2%)	
**Total**	**4604 (100.0%)**	**904 (19.6%)**		**2823 (100.0**%**)**	**360 (12.8%)**	
**Rounds of MDA** ^**c**^			**< 0.001** ^**d**^			**< 0.001** ^**d**^
Not taken MDA	738 (16.0%)	101 (13.7%)		198 (7.0%)	14 (7.1%)	
1–4 rounds	2422 (52.6%)	497 (20.5%)		1191 (42.2%)	131 (11.0%)	
≥ 5 rounds	1444 (31.4%)	306 (21.2%)		1434 (50.8%)	215 (15.0%)	
**Total**	**4604 (100%)**	**904 (19.6%)**		**2823 (100.0**%**)**	**360 (12.8%)**	
**Sub-districts** ^**c**^			**< 0.001** ^**d**^			
Manyoro	815 (17.7%)	212 (26.0%)				
Pungu	1951 (42.4%)	384 (19.7%)				
Navrongo-East	1838 (39.9%)	308 (16.8%)				
Kongo-Pitanga				582 (20.6%)	63 (10.8%)	**< 0.001** ^**d**^
Pelungu				650 (23.0%)	112 (17.2%)	
Sakote				646 (22.9%)	79 (12.2%)	
Nagodi				539 (19.1%)	61 (11.3%)	
Zanlerigu				406 (14.4%)	45 (11.1%)	
**Total**	**4604 (100.0%)**	**904 (19.6%)**		**2823 (100.0**%**)**	**360 (12.8%)**	

^a^ median (interquartile range)

^b^ Mann-Whitney U test

^e^ Cochran-Armitage test for trend

^c^ total number (%)

^d^ Pearson-Chi-square

**Table 2:** MDA rounds and FTS prevalence among age and years lived in endemic area of study participants.

Variable	KNEM FTS-CFA POSTIVE (N = 904/4604)				NABDAM FTS-CFA POSTIVE (N = 360/2823)			
	MDA ROUNDS				MDA ROUNDS			
	0 round	1–4 rounds	≥ 5 rounds	p-value	0 round	1–4 rounds	≥ 5 rounds	p-value
**Age groups (years)**	**Total screened/FTS-CFA positive (prevalence)**				**Total screened/FTS-CFA positive (prevalence)**			
5-<10	278/7 (2.5)	38/2 (5.3)	2/1 (50.0)	**<0.001** *	0/0 (0.0)	2/1 (50.0)	0/0 (0.0)	**<0.001** *
11_20	97/7 (7.2)	540/42 (7.8)	71/6 (8.5)		42/1 (2.4)	320/18 (5.6)	68/10 (14.7)	
21–30	88/12 (13.6)	522/113 (21.6)	182/30 (16.5)		50/4 (8.0)	326/25 (7.7)	201/39 (19.4)	
31–40	91/22 (24.2)	430/95 (22.1)	251/50 (19.9)		44/2 (4.5)	237/32 (13.5)	267/36 (13.5)	
41–50	75/22 (29.3)	355/90 (25.4)	315/63 (20.0)		25/3 (12.0)	122/23 (18.9)	318/49 (15.4)	
51–60	51/14 (27.5)	259/78 (30.1)	238/57 (24.0)		18/1 (5.6)	94/18 (19.1)	286/50 (17.5)	
>60	58/14 (24.1)	278/77 (27.7)	385/100 (26.0)		19/3 (15.8)	90/15 (16.7)	294/31 (10.5)	
**Total**	**738/101 (13.7)**	**2422/497 (20.5)**	**1444/306 (21.2)**		**198/14 (7.1)**	**1191/131 (11.0)**	**1434/215 (15.0)**	
**Years lived in endemic area**								
2-<10	308/12 (3.9)	86/9 (10.5)	8/1 (12.5)	**<0.001** *	30/0 (0.0)	22/0 (0)	2/1 (50.0)	**<0.001** *
11_20	127/10 (7.9)	600/53 (8.8)	97/10 (10.3)		39/0 (0)	341/9 (2.6)	48/2 (4.2)	
21–30	94/13 (13.8)	545/120 (22.0)	223/38 (17.0)		41/1 (2.4)	284/19 (6.7)	153/14 (9.1)	
31–40	92/25 (27.2)	424/100 (23.6)	281/56 (20.0)		26/2 (7.7)	161/22 (13.7)	222/15 (6.8)	
>40	117/41 (35.0)	767/215 (28.0)	835/201 (24.1)		36/5 (13.9)	210/33 (15.7)	666/65 (9.8)	
**Total**	**738/101 (13.7)**	**2422/497 (20.5)**	**1444/306 (21.2)**		**172/8 (4.7)**	**1018/83 (8.2)**	**1091/97 (8.9)**	

*Cochran-Armitage test for trend; Statistical significance (p<0.05); 542 missing data for years lived in endemic area for Nabdam district

In KNEM, 35.3% and 24.1% of all female and male participants respectively had taken ≥5 MDA rounds. In Nabdam, more than half (52.9%) of all female participants had taken ≥5 MDA rounds (Fig 1). Though the participants had lived in the endemic communities for close to or 35 years, only 31.4%/50.8% had taken ≥5 rounds of MDA in KNEM/Nabdam respectively (Table 1).

In Nabdam district, a total of 2849 participants were recruited and screened for CFA using the FTS. However, 2823 participants (with 62.5% females) with a median age of 38.0 were used in the analysis due to incomplete data from 26 participants. Men were mostly infected (15.6% vs. 11.1%; p = 0.001). A total of 360 (12.8%) were FTS positives, with the highest FTS-CFA prevalence recorded in the 51-60-year-olds age group (17.3%) (Table 1). In total, 1264 (17%) FTS positives were recorded in the study from a total of 7427 screened in both districts.

There was a statistical difference (p<0.001) between age and the number of treatment rounds received regarding CFA prevalence. Generally, in the two districts, CFA prevalence significantly increased (p<0.001) with a longer duration of stay in the endemic area and MDA rounds received (Table 2). A total of 69% of the participants in KNEM and 50.8% in Nabdam reported taking <5 MDA treatment rounds (Table 1). Thus, as the treatment rounds increased, the number of participants who participated reduced.

### Bi- and multivariate modelling analyses for LF infection in KNEM and Nabdam districts

Binary logistic regression analyses (modelling) were done for gender, age, years lived in endemic area, district and number of IVM rounds intake to determine the variables fit to be included in the multivariate model. From the bivariate model, all variables included showed statistically significant associations (p<0.05) with FTS positivity (Table 3). In the multivariate model to determine the factors that exclusively and independently predicted the odds of an individual being FTS-CFA positive—all variables (age, gender, years lived in endemic area and the district), except MDA rounds stood out as independent predictors for FTS-CFA positivity. A male individual was 1.5 times more likely to be FTS positive as compared to a female [AOR = 1.43, CI = 1.25–1.62]. Also, the odds of getting infected increased as one aged, with the age group of 51–60 years having 12 times increased odds of being FTS positive compared to the 5-≤10 age group [AOR = 12.13, CI = 6.01–24.48]. An individual from KNEM was almost twice likely to be FTS positive compared to an individual from Nabdam [AOR = 1.85, CI = 1.61–2.12]. There was no significant association (p>0.05) between MDA rounds taken and FTS-CFA positivity. Participants that consumed MDA rounds 1–4 and ≥5 had the same odds to be infected as those who have never taken ivermectin (Table 3).

**Table 3:** Bivariate and multivariate modelling analyses for FTS positivity in study districts.

Variable	COR (95% CI)	p-value	AOR (95% CI)	p-value
**Age Group (years)**				
5-≤10	1		1	
11–20	2.75 (1.37–5.54)	0.005	2.88 (1.40–5.91)	0.004
21–30	6.72 (3.41–13.25)	<0.0001	7.59 (3.77–15.28)	<0.0001
31–40	7.56 (3.84–14.89)	<0.0001	8.72 (4.34–17.54)	<0.0001
41–50	9.00 (4.57–17.71)	<0.0001	10.32 (5.13–20.76)	<0.0001
51–60	10.35 (5.24–20.42)	<0.0001	12.13 (6.01–24.48)	<0.0001
>60	9.53 (4.84–18.77)	<0.0001	10.90 (5.41–21.97)	<0.0001
**Gender**				
Male	1.25 (1.10–1.41)	0.001	1.43 (1.25–1.62)	<0.0001
Female	1		1	
**Rounds of MDA**				
Not taken MDA	1		1	
1–4 rounds	1.50 (1.21–1.86)	<0.0001	1.23 (0.98–1.54)	0.077
≥ 5 rounds	1.58 (1.27–1.96)	<0.0001	1.13 (0.90–1.44)	0.297
**District**				
Nabdam	1		1	
KNEM	1.67 (1.46–1.91)	<0.0001	1.85 (1.61–2.12)	<0.0001

COR, Crude Odds Ratio; AOR, Adjusted Odds Ratio; CI, Confidence interval

**Fig 1A**. Percentage of MDA intake significantly differed with gender in both districts (p<0.0001; p<0.002). However, regarding FTS-CFA prevalence, there was a significant difference between number of MDA rounds received among males and females in KNEM (p<0.0001) but not in Nabdam (p = 0.202) (**Fig 1B).**

### Reasons for participants MDA intake failure in both districts

**Fig 2:**
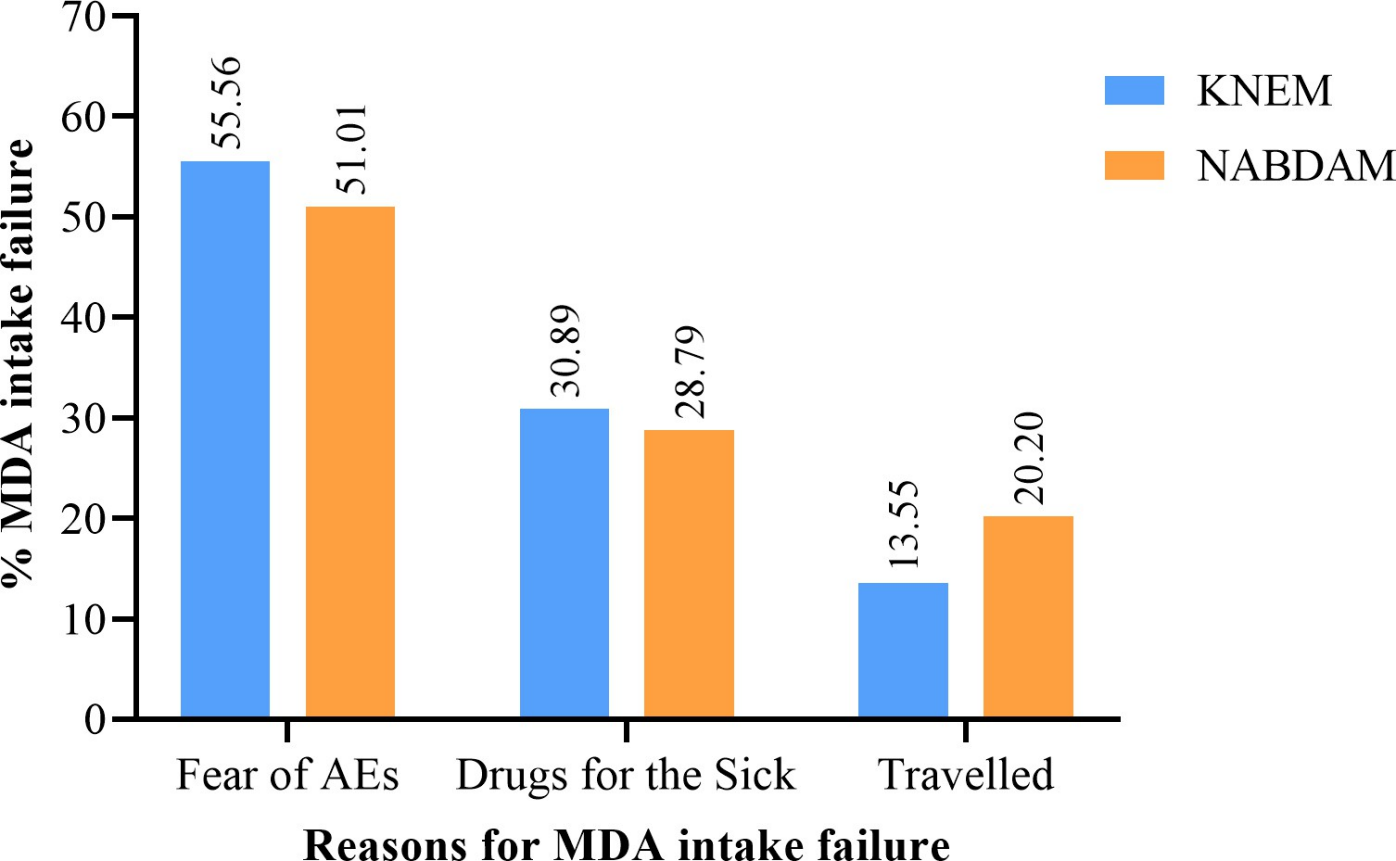
Cited reasons contributing to null MDA intake among participants in both districts.

A total of 12.6% [936—(KNEM: N = 738 (16.0%); Nabdam: N = 198 (7.0%))] had never taken part in the annual MDA programme since its inception. Out of the 738 participants who had never taken part in KNEM, 410 (55.56%) reported being afraid of adverse events (AEs) that may come along with the MDA which might make them ineffective for their farm activities. Also, 228 (30.9%) posited that the drugs are meant for “sick people”. However, 100 (13.6%) said they had lived infrequently in the municipality, mostly travelling to the southern part of the country or to the neighbouring countries, most notably Burkina Faso. They would then only return during the harvest season, by which time the MDA distribution would have ended (Fig 2). Similar reasons were cited by the 198 (7.0%) participants from the Nabdam district. More than half (101, 51.0%) reported being afraid of AEs, with 57 (28.8) and 40 (20.2%) respectively stating that the MDA drugs are meant for sick people and travelling in-and-out of the district at the time of the community MDA (Fig 2).

Generally, the LE affected participants increased with age. None of the participants present for the screening in the Nabdam district had LF-related pathology(-ies).

### Prevalence of MF and the demographic distribution of study participants in both Districts

A total of 799 and 210 participants from KNEM and Nabdam respectively had venous night blood samples randomly collected for MF assessment. Of the 799/210 participants, a total of 694/191 were FTS positives whiles 105/19 were FTS negatives in KNEM/Nabdam. Out of the 694 and 191 FTS positives, only 5 (0.7%) and 1 (0.5%) were positive for MF in KNEM and Nabdam, respectively. Three out of the total 6 MF positives had Sedgewick and Nucleopore counts <5Mf/mL, with the remaining 3 having MF count of 5-<100Mf/mL (Table 4). Males recorded MF positivity in 2 out of 246 cases (0.8%) whiles females recorded 3 out of 448 cases in the KNEM (0.7%). In Nabdam, MF positivity of 1, a male, out of 191 (0.5%) was recorded (Table 5). As the number of MDA rounds increased, there was a general reduction in MF positivity (Table 5). None of the 105/19 (KNEM/Nabdam) FTS-negative participants had MF in the night blood. Assessment of MF could not be done in children ≤10 years due to several challenges (This has been elaborated on in the discussion section). It must be noted that out of the 19.6% and 12.8% FTS positive individuals from KNEM and Nabdam respectively, the MF positivity was 0.7% and 0.5% respectively. Thus, the MF positives would be expected to be close to one fifth of 0.7% (0.14%) in KNEM and 0.128 times the 0.5% MF (0.06%) in Nabdam, had all the 4604/2823 (KNEM/Nabdam) been sampled at night for MF screening.

**Table 4:** Sedgewick and nucleopore counts of MF positive participants in KNEM and Nabdam.

PID	Age	Gender	MDA rounds	Years lived in end. area	District	MF assessment/count	
						Sedgewick (Mf/200μL of blood)	Nucleopore (Mf/mL of blood)
085–204	32	M	1	32	KNEM	0	1
076–254	48	F	3	48	KNEM	1	1
001–501	55	M	1	36	KNEM	5	15
005–506	45	F	6	45	KNEM	11	85
016–501	58	F	10	50	KNEM	0	3
023–089	42	M	3	39	Nabdam	11	54

PID—Participant identification number

**Table 5:** Demographic distribution and MF prevalence among FTS-positive participants in KNEM and Nabdam Districts.

Characteristic	Screened		MF Positives		p-Value	
	KNEM	Nabdam	KNEM	Nabdam	KNEM	Nabdam
**Age (years)** ^a^	38.0 (31)	38.0 (29)				
**Years lived in endemic community** ^a^	34.0 (30)	35.0 (27)				
**Gender** ^b^					**0.081** ^**c**^	**0.673** ^**c**^
Female	448 (64.6)	104 (54.5)	3 (0.7)	0 (0.0)		
Male	246 (35.4)	87 (45.5)	2 (0.8)	1 (1.1)		
**Total**	**694 (100.0)**	**191(100.0)**	**5 (0.7)**	**1 (0.5)**		
**Age group (years)** ^b^					**0.650** ^**d**^	**0.825** ^**d**^
10–19	30 (4.3)	26 (13.6)	0 (0.0)	0 (0.0)		
20–29	58 (9.2)	44 (23.0)	0 (0.0)	0 (0.0)		
30–39	123 (17.7)	42 (22.0)	1 (0.8)	0 (0.0)		
40–49	143 (20.6)	36 (18.8)	2 (1.4)	1(2.8)		
50+	340 (49.0)	43 (22.5)	2 (0.6)	0 (0.0)		
**Total**	**694 (100)**	**191(100)**	**5 (0.7)**	**1 (0.5)**		
**Rounds of MDA** ^**b**^						
0	67 (9.7)	7 (3.7)	0 (0.0)	0 (0.0)	**0.604** ^**c**^	**0.521** ^**c**^
1–4	358 (51.6)	73 (38.2)	3 (0.8)	1 (1.4)		
5+	269 (38.8)	111 (58.1)	2 (0.7)	0 (0.0)		
**Total**	**694 (100)**	**191 (100)**	**5 (0.7)**	**1 (0.5)**		
**Sub-districts** ^b^					**0.052** ^**c**^	**0.231** ^**c**^
Manyoro	138 (19.9)	-	1 (0.7)	**-**		
Navrongo-East	159 (22.9)	-	2 (1.3)	**-**		
Pungu	397 (57.2)	-	2 (0.5)	**-**		
Zanlerigu + Nagodi	-	73 (38.2)	-	0 (0.0)		
Kongo-Pitanga	-	27 (14.1)	-	1 (3.7)		
Sakote + Pelungu	-	91 (47.6)	-	0 (0.0)		
**Total**	**694 (100)**	**191 (100)**	**5 (0.7)**	**1 (0.5)**		

^a^ median (interquartile range)

^b^ total number (%)

^c^ Pearson-Chi-square

^d^ Cochran-Armitage test for trend

### Og4C3 antigen assessment using Tropbio ELISA

A total of 799/210 participants (KNEM/Nabdam) were randomly selected for Og4C3 CFA assessment using Tropbio ELISA; out of which 694/191 (KNEM/Nabdam; total of 885) were FTS positives. Assessment of Og4C3 antigen could not be done in children <10 years due to challenges in night blood sampling (see discussion session). Of these 694/191 (KNEM/Nabdam), only 157 (22.6%)/44 (23.0%) were positive (22.7%; 201 out of 885 tested) in TropBio, with varying degrees of antigenemia. As Og4C3 antigen concentration decreased (from high to low), the number of positive participants increased in each district. Thus, most of the positives have low Og4C3 antigen concentrations. Majority (≥77.0%) of the FTS-positive participants were negative for the Og4C3 CFA in each district. All 6 MF-positives in the two districts were all also high Og4C3 antigen positives. Again, all the 105/19 (KNEM/Nabdam) FTS-negative participants were also Og4C3 antigen negative (Table 6).

From a total number of 7427 (4604/2823) screened and analysed participants in KNEM/Nabdam, 1264 (17%; 904/360) were FTS positive and 6163 (83%; 3700/2463) were FTS negative. Out of the 1264 FTS-positives 885 (70%; 694/191) and out of the 6163 FTS negatives 124 (2%; 105/19) were randomly selected for the Og4C3 antigen assessment. Because of the unbalanced data set from the screening data; calculation was done back to the original sample size with the following assumptions: i. all FTS negatives are also negative for the Og4C3 antigen assessment, ii. 22.7% (201 Og4C3 positives out of 885 FTS positives) of the FTS-positives are also Og4C3 positive. Taking these assumptions into consideration, 287 Og4C3 positives (22.7% of 1264 total FTS positives) can be estimated and this can be related to the total of 7427 screened participants which results in an estimated Og4C3 positivity rate of 3.8% for both districts together. That is, if Og4C3 positivity of 22.7% was recorded from 17% FTS positives, then a total of 3.8% Og4C3 positivity will be recorded had all the 7427 were sampled and screened for Og4C3.

**Table 6:** Og4C3 antigen Tropbio ELISA results from FTS-Positive/Negative participants.

	Og4C3 antigen Tropbio ELISA				Total
	Negative	Low positive	Medium positive	High positive	
**FTS status (KNEM)**					
694 randomly selected FTS-positive participants	537 (77.4%)	126 (18.2%)	16 (2.3%)	15 (2.2%)	**694**
105 randomly selected FTS-negative participants	105 (100.0%)	0 (0.0%)	0 (0.0%)	0 (0.0%)	**105**
**Total**	**642**	**126**	**16**	**15**	**799**
**FTS status (Nabdam)**					
191 randomly selected FTS-positive participants	147 (77.0%)	37 (19.4%)	4 (2.1%)	3 (1.6%)	**191**
19 randomly selected FTS-negative participants	19 (100.0%)	0 (0.0%)	0 (0.0%)	0 (0.0%)	**19**
**Total**	**166**	**37**	**4**	**3**	**210**

### Distribution of lymphedema and hydrocele among age and gender of participants in KNEM

**Fig 3:**
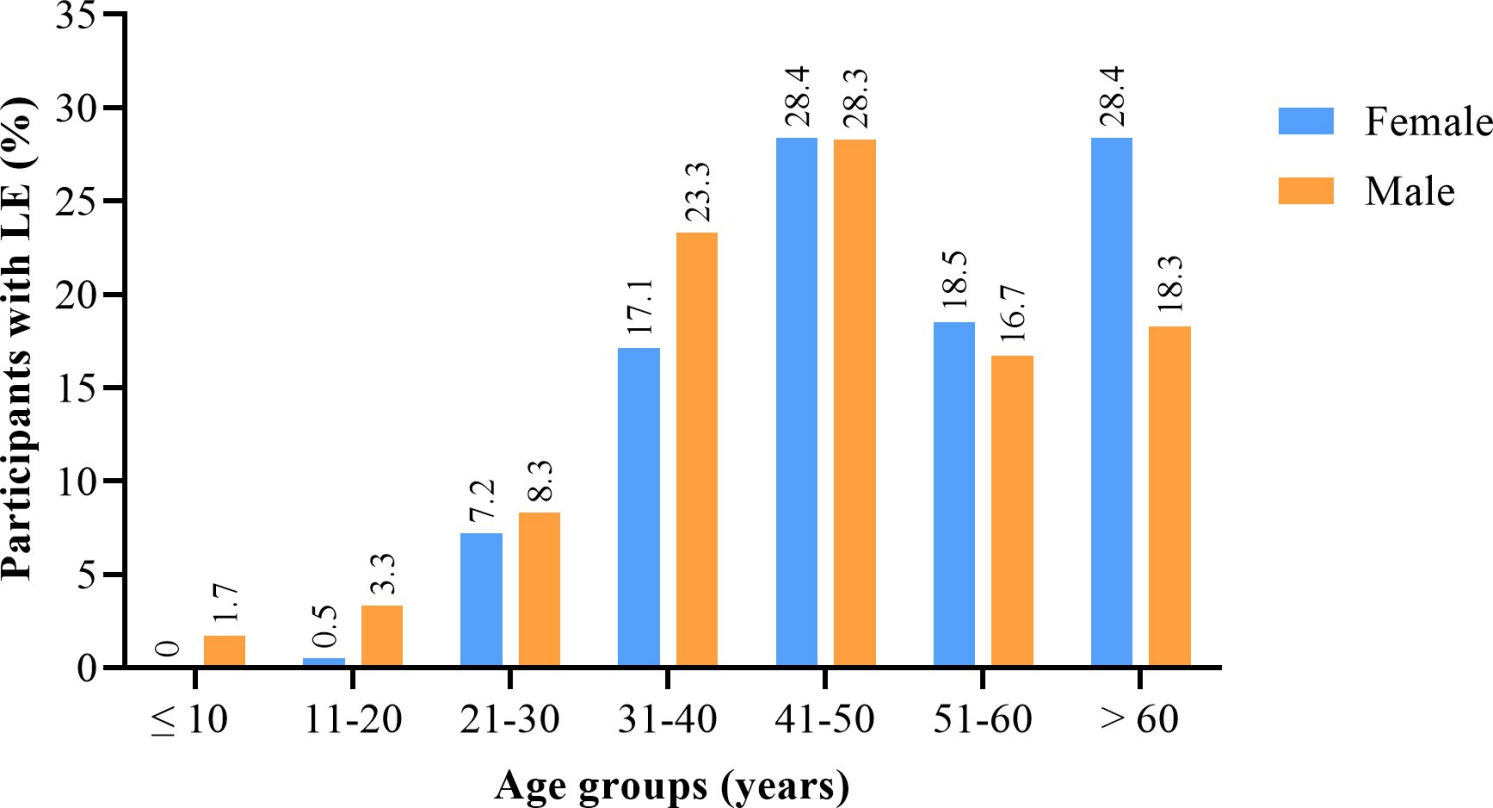
Age and gender distribution of participants with filarial lymphedema in KNEM.

Of the 4604 participants screened in KNEM, 328 (7%) presented with clinical manifestations of LF. The majority (282, 86.0%) were lymphedema. A total of 220/2997 (7.3%) women screened had lymphedema. Among the males, 62/1607 had lymphedema (3.9%) and 46/1607 (2.9%) had cases of hydrocele. The majority of the pathology participants (172/282, 61%) had mild LE (stages 1–2). Participants with stages 3–4, classified as moderate LE accounted for 16% (45/282) whiles stages 5–7 classified as severe LE accounted for 12.4% (35/282). More than half (52.1%) of the pathology participants were from the Pungu sub-district (S1 Table). No cases of LE were identified among participants who were less than 10 years old. The prevalence of LE rose steadily from the age ≤10 years, peaking in the age group 41–50 years in both males and females. The LE cases declined and rose again in >60 years age group (Fig 3). Among the participants screened in Nabdam, none came presenting with any case of LE or hydrocele (S1 Table).

## Discussion

The implementation of ivermectin and albendazole as MDA started in the two hotspot districts, KNEM and Nabdam, in 2000 and 2005, respectively [7]. While MDA is still ongoing in the Nabdam district, the programme was stopped 5 years ago in KNEM. Considering how long the MDA programme has run in the two districts, transmission is expected to be interrupted [17]. In this present study, the overall prevalence of CFA assessed by the Alere FTS was 19.6%/12.8% (KNEM/Nabdam). With an overall projected Og4C3 antigen prevalence of 3.8% from the two districts, the figure exceeds the WHO 2% antigenemia prevalence threshold that is required for LF elimination [17,18]. An individual from KNEM was almost twice likely to be FTS positive compared to an individual from Nabdam. This might be due to KNEM having had high baseline LF infection prevalence compared to Nabdam [7]. Reports suggest high LF baseline prevalence and the usage of the FTS in transmission assessment increases the risk of pre-TAS failure [5].

It is interesting to observe that in the KNEM, MF has been reduced from 29.6% at baseline [7] in the year 2000 to the current 0.7% among the CFA positives in this study. Considering that all the 105/19 FTS-negative participants assessed for MF were negative, it is estimated that the 0.7%/0.5% MF positivity would have been 0.14%/0.06% (KNEM/Nabdam), which is by far <1% threshold had all the recruited participants been screened for MF. This, however, would mean that at least one of the two pre-TAS criteria; that is having MF positivity <1% has been fulfilled in the two hotspot districts, denoting a halt in the transmission of the *W*. *bancrofti* infection.

There is the need to do a bigger study to include more if not all infected and non-infected in the MF and Og4C3 CFA assessment. Considering that ≥77% of the FTS positive participants from each district tested negative for the Og4C3 antigen, and the fact that majority (81.1%) of the ELISA positives had low Og4C3 antigen concentrations; it can be deduced that the FTS is more sensitive in detecting residual circulating filarial antigens in the blood since residual target antigens are slowly cleared from circulation, as reported by others [10, 11, 19, 20]. This proposition is further strengthened by the fact that the MF positives were clearly <1% in each district.

The FTS, as other studies have already revealed is more sensitive [21] as has also been seen in this study. This is supported by the fact that in 2007, the CFA prevalence as determined by the ICT card was 6.6% in KNEM [7]. Though the FTS is relatively sensitive and superior to the ICT for mapping, for transmission assessment surveys, and for post-MDA surveillance [3, 22], ⁓200% increase in antigenemia prevalence from the 6.6% in 2007 to the current 19.6% in this study as determined by the FTS is very high. Reader error, inability to get enough capillary blood from fingerprick, faulty batch of test kits as well as the Og4C3 ELISA having limited sensitivity at the revised cutoffs—necessitating the need for a new cut off point in this end game period, could have accounted for the striking difference in the FTS and Og4C3 ELISA positives as reported by Gass et al., [23]. Nevertheless, for the first two reasons, competent and trained personnel were used so they could be ruled out. However, this may also be due to the FTS’s inadequate specificity for antigens from other filarial nematodes such as *M*. *perstans*, *O*. *volvulus* and *L*. *loa*—leading to antigenic cross-reactivity with that of *W*. *bancrofti* and subsequently false positives as reported by others [10,11,24].

Recent studies have suggested that although *M*. *perstans* do not cause lymphedema, they can result in false positives on FTS due to antigenic cross-reactivity with *W*. *bancrofti* [10]. Further to this, some FTS positive cases in the referenced study [10] harboured *M*. *perstans* MF, rather than *W*. *bancrofti*, with negative qPCR results for *W*. *bancrofti*. Dolo (2020), also reported that the likelihood of being *W*. *bancrofti* infected using the FTS was higher in individuals with detectable *M*. *perstans* MF, despite the fact that these infections are transmitted by different vectors [24].

Also, in a study which integrated multiple biomarkers to increase the sensitivity for the detection of *O*. *volvulus* infection—a preliminary screening using IgG4 (an antibody isotype known to provide antigenic specificity) indicated significant cross-reactivity of OVOC11950 and OVCO10602 with sera from subjects with *Wuchereria bancrofti* and/or *L*. *loa* infections [11]. Thus, RDTs for *W*. *bancrofti* detection likely faces problem with diagnostic specificity.

Ghana is endemic for *O*. *volvulus* [12] and *M*. *perstans* [13,14]. The high endemicity of onchocerciasis in the Upper East region of Ghana led the former OCP to focus on onchocerciasis in the northern and uppermost parts of the country, which are well known for cases of blinding onchocerciasis [12]. Bawku West, Kassena Nankana West and Builsa North—the adjoining districts to Nabdam and KNEM (the two hotspot districts surveyed in the current study) are still endemic for onchocerciasis with the infection still persisting [12,15]. Thus, some of the FTS positives in the current study may likely be due to *Onchocerca* and/or *Mansonella* infection rather than *W*. *bancrofti*. The findings from the previous studies [10,11,24] signify the importance of the use of more rigorously specific tests for LF in areas where *O*. *volvulus* and *M*. *perstans* are present. Subsequently, the striking difference between the FTS and Og4C3 ELISA which may be due to false positive FTS serological testing caused by cross-reactivities of *W*. *bancrofti* with *Onchocerca* and/or *Mansonella* antigens is important for the Ghana LF elimination program, as this may be useful for treatment monitoring, surveillance, pre-TAS and TAS.

More studies on the sensitivity and specificity of the FTS and the Og4C3 ELISA, like the one conducted by Chesnais *et al* [21] need to be done to ascertain the best diagnostic tool for monitoring and post-treatment surveillance. Scoring the positive test bands in the FTS semi-quantitatively as weak, equal in density (vrs the control) and deeper and then determining the antigenemia level of such different bands with ELISA will further help elucidate the sensitivity of the FTS as done by Chesnais *et al*. [21].

As reported by other studies, most sentinel sites showed characteristically low MF prevalence but relatively higher antigen prevalence after several rounds of MDA [6]. The same finding was observed in this study and has also been reported in other prevalence studies [25]. This calls for a revision/adjustment of the long-standing stringent 2% CFA threshold especially after more sensitive diagnostic kits such as the FTS have been introduced for post-treatment surveillance [1,3]. Notably, even the relatively less sensitive Og4C3 spun out a prevalence >2% in this study and this may indicate that the <2% CFA threshold needed to pass pre-TAS may be too stringent. The revision and subsequent validation of the <2% CFA threshold by WHO as was also recommended elsewhere [6] may help prevent further treatment rounds which might not be needed.

Though arithmetic and statistical projections bring the MF prevalence in the two hotspot districts to far <1.0%, effort should be made by the regional and district health and NTD management to reduce CFA load to the current < 2.0% threshold. This is because if not checked, there could be recrudescence. Thus, new strategies to clear the remaining active infection (0.7%/0.5%) before it spreads to nearby “LF free” communities should be implemented to eliminate LF from the district [26]. There are three ways to do this; (1) either to continue treating with the current MDA drugs for more years, or (2) to actively “test and treat” infected people with alternative drugs such as doxycycline as is being currently done in OEPA [27], or (3) to treat infected people with the new triple drugs regimen of ivermectin, DEC and albendazole (IDA) [28].

Option (1) may not be the prudent choice due to persistent non-compliance and lower response from a section of the people to the current MDA drugs and therefore may still have MF in the blood [29]. The second option—will rather ensure a definite impact of control on the reservoir of the parasite and possibly achieve its elimination. Our ongoing trial under the TAKeOFF project, “test and treat for regional elimination of LF” [30] will shed more light on this option.

It is worth noting that the single MF positive person in Nabdam was promptly treated with ivermectin/albendazole and 100mg/day of doxycycline for 4 weeks to further kill the female adult worms to ensure he does not transmit the infection to others in the community. There is also the need to follow those who were FTS+MF- to determine how long the residual filarial antigen takes to be cleared from the blood of the host, or whether they become again MF positive.

Contrary to studies done by Lich (2012) in which males and females were found to be equally susceptible to filarial infections [31], in this study and as has also been reported in other studies [32,33], it was observed that there was a statistically significant higher CFA prevalence among males than females in both hotspot districts (p = 0.033 and p = 0.001). A male individual was 1.5 times more likely to be FTS positive as compared to a female. Though, the study had considerably more women than men in this study, the disparity in CFA prevalence regarding gender might be attributed to men generally engaging in more outdoor social activities even at night and farming along the banks of dams and dugouts all day as was observed in the study communities. Thus, the men may have had an increased risk of exposure to infective mosquito bites [34–36] and may as well miss some treatment rounds as 17.2% of all male participants had never taken ivermectin.

Generally, in the two districts, FTS-CFA prevalence significantly increased with longer duration of stay in the endemic area and MDA rounds received. This was however not surprising as ⁓ ≥50% (69% in KNEM) of the participants in both districts reported to have taken <5 treatment rounds, though the participants had stayed in the endemic communities for years similar to their mean ages in both districts. Thus, the number of participants reduced as the treatment rounds increased. This may allude to the fact that the participants showed apathy towards the continual intake of the MDA drugs probably due to treatment fatigue. There was no significant association (p>0.05) however between MDA rounds taken and FTS-CFA prevalence, with participants consuming 1–4 and ≥5 MDA rounds having same odds of being infected as those who have never taken IVM.

In both districts, a smaller prevalence was found in the younger age groups and the prevalence increased significantly with age (p<0.001). The odds of getting infected increased as one aged as has also been reported by others [34,35]. The age group of 51–60 years had 12 times increased odds of being FTS positive compared to the 5-≤10 age group. Thus, as one continues to stay in the endemic area, the person is likely to be exposed to several infection bites from mosquitoes, yet may show apathy and fatigue towards continual MDA treatment. The MDA programme is geared towards reducing and halting new LF infection transmission [37,38]. Therefore, for children who were born after ≥5 effective MDA rounds, LF prevalence is expected to be zero, or more so <2% if the transmission is halted [39]. However, in KNEM, CFA prevalence in children ≤10 years was 2.8%, and this may point to the presence of ongoing low-level transmission, though further assessment is needed to ascertain this. Other studies among children in Ghana and elsewhere reported similarly, where CFA prevalence exceeded the <2% WHO threshold [40,41]. The infected children in the present study should also have been identified and assessed for MF. However, this was a challenge as parents were unwilling to allow night blood sampling of their children (10 pm-12 am), as in most cases they would be sleeping. Also, parents in most cases were repulsive and labelled the research team as “night blood suckers”. However, this challenge is not unique to this study as there have been instances in India where night surveys have been met with repulsion with scientists being labelled as ‘vampires’ and ’bloodsuckers’ [42,43].

Seasonal migration is likely to have aided in the very high CFA prevalence in Manyoro (in KNEM), a border sub-district located near the South of Burkina Faso serving as a hub of extensive migration and commercial activities between the two countries. This poses a problem during MDA and subsequent assessment of the LF infection [44]. The individuals in the study districts normally travel out during and after planting and only return during the harvest season, by which time the MDA distribution would have ended. Thus, infected people who are frequent travellers never get treated. The frequent travellers who are also treated may get reinfected in other endemic areas, especially in Burkina Faso. Burkina Faso currently has about 5 million people at risk of infection compared to Ghana’s estimated 1 million, having undergone ≥10 years of MDA with persistent low-level transmission [1]. MDA should be intensified at the border towns which may serve as reservoirs for recrudescence and transmission in already “LF-free” areas.

According to the community and district MDA register, MDA compliance has been okay—consistent with our findings as 88.5% of the study participants had taken the MDA at least once. However, compliance to the drug consumption reduced as the number of MDA rounds increased. Out of the 7453 recruited participants, 12.6% had never taken part in the annual MDA. Prominent among the reasons cited for the unwillingness to partake in the MDA, was the fear of experiencing adverse events such as feverishness, diarrhoea and physical discomfort which results from the death of the MFs—projecting that will make them ineffective for their farm activities [45–47]. Assessment of drug coverage (total population to whom the drugs are delivered to by the community health volunteers) will also help reveal the true impact of MDA as this may not be an accurate reflection of drug compliance (the actual number of people consuming the drug) [44,48]. This was however not done in this study. Using the community MDA register for households, a more focused and intensified MDA can be undertaken—where historically never treated individuals, seasonal migrants and systematically non-compliers should be specifically targeted to have MF killed if any is actively infected. This will prevent recrudescence.

In a rural setting such as the KNEM and Nabdam characterised by comparatively lower socio-economic status, sparse distribution of houses also makes it difficult for the distribution of MDA to reach remote areas of the community [34]. Community health volunteers (CHVs) may not be motivated enough to provide the best drug distribution services. Informal discussions with the CHVs revealed that in as much as they know the benefit of the services they provide, incentives in the form of structured remuneration, and the provision of basic equipment such as boots and torchlights will boost their morale as observed in examples set by Mali [49].

Looking at the number of lymphedema patients in the districts, especially in the KNEM, there is the need for the WHO morbidity management plan to be strengthened in the districts. Hydrocelectomy is organized from time to time in these districts by national hydrocelectomy groups, NGOs, etc., but a management plan for lymphedema patients had just begun in the study districts. Our group together with the regional and district NTD programme recently organised a workshop on hygiene-based lymphedema morbidity management for health workers in the study districts to further enlighten them on the disease management [50].

## Conclusions

Not only the FTS, but also the lower Og4C3 antigen prevalence in the study districts exceeded the WHO pre-TAS recommended <2% antigenemia threshold required for elimination. This may point to the fact that there may still be some degree of transmission, although the stringent long-standing <2% CFA threshold may need revision and validation especially after more sensitive diagnostic kits such as the FTS have been introduced for post-treatment surveillance. Projections from the MF results however reveal a halt in LF transmission in the two hotspot districts. More studies need to be done on the sensitivity and specificity of the current LF diagnostic tools available for post-treatment surveillance as antigenic cross-reactions with other filarial nematodes may be problematic. From the treatment coverage recorded so far, the National NTD programme is doing well to eliminate LF from Ghana. However, MDA should be more focused on seasonal non-compliers and intensified at the border towns, and in addition employ and implement alternative treatment approaches such as test and treat strategy so that instead of distributing ivermectin and albendazole to all people at risk, doxycycline can be administered to MF or CFA positive inhabitants.

## Supporting information

S1 Table The distribution of LF pathology cases among sub-districts in KNEM. (DOCX)
