# Supplementary material for: Occurrence of Lymphatic Filariasis infection after 15 years of mass drug administration in two hotspot districts in the Upper East Region of Ghana
Source: PLoS Negl Trop Dis. 2022 Aug 4;16(8):e0010129. doi: 10.1371/journal.pntd.0010129 (PMC9380951; doi:10.1371/journal.pntd.0010129)
Supplement: S1 Table — (DOCX) [file pntd.0010129.s001.docx]

**S1 Table: The distribution of LF pathology cases among sub-districts in KNEM**

|  | **SUB-DISTRICTS** | | |  |
| --- | --- | --- | --- | --- |
|  | **Number of pathology cases (%)** | | | **Total** (%) |
|  | **Pungu** | **Navrongo East** | **Manyoro** |  |
| **LF PATHOLOGY** |  |  |  |  |
| Lymphedema (LE) | 144 (51.1%) | 83 (29.4%) | 55 (19.5%) | 282 (86.0%) |
| Hydrocele (HYD)  **Total** | 19 (41.3%)  **163 (49.7%)** | 9 (19.6%)  **92 (28.0%)** | 18 (39.1%)  **73 (22.3%)** | 46 (14.0%)  **328 (100.0%)** |
